# Repression of YAP by NCTD disrupts NSCLC progression

**DOI:** 10.18632/oncotarget.13668

**Published:** 2016-11-26

**Authors:** Jiwei Guo, Yan Wu, Lijuan Yang, Jing Du, Kaikai Gong, Weiwei Chen, Juanjuan Dai, XueLin Li, Sichuan Xi

**Affiliations:** ^1^ Cancer Research Institute, Binzhou Medical University Hospital, Binzhou 256603, P.R. China

**Keywords:** lung cancer, YAP, NCTD, EMT, cell cycle arrest

## Abstract

The efficacy of available lung cancer therapeutic interference is significantly limited by various resistance mechanisms to those drugs. Activation of the oncogene YAP underlying the initiation, progression, and metastasis of lung cancer associates with poor prognosis and confers drug resistance against targeted therapy. In this study, we evaluated the specificity of norcantharidin (NCTD) in repressing YAP to inhibit non-small cell lung carcinoma (NSCLC) progression. Our study revealed that YAP signal pathways were aberrantly activated in lung cancer tissues and cells which rendered more proliferative and invasive phenotypes to human lung cancer cells. We confirmed that NCTD specifically repressed YAP signaling pathway to interfere the YAP-mediated non-small cell lung carcinoma progression and metastasis via arresting cell cycle, enhancing apoptosis and inducing senescence. We also found NCTD-mediated repression of YAP decreased epithelial-to-mesenchymal transition (EMT) and reduced the motile and invasive cellular phenotype *in vitro* via enhancing E-cadherin and decreasing fibronectin/vimentin. Mechanistic investigations revealed that NCTD transcriptionally downregulated YAP and post-translationally modulated the subcellular redistribution of YAP between nucleus and cytoplasm. Collectively, our results indicated that NCTD is a novel therapeutic drug candidate for NSCLC which specifically and sensitively target YAP signal pathway.

## INTRODUCTION

As the number one cancer killer worldwide [1–6] and with enormous efforts in surveillance, surgery, radiotherapy and platinum-based chemotherapy, lung cancer still remains the most aggressive malignant tumor with one of the lowest survival rates [7, 8, 9]. The new approaches to detect, cure, and prevent this malignant disease are currently in emergent demands.

One of critical challenges for targeted lung cancer chemotherapies is rapid and unavoidable drug resistance development. Aberrant activation Mst/Yap pathways has been reported to be involved in drug resistance acquired from treatment with cisplatin and erlotinib (EGFR inhibitor) in non-small cell lung cancer (NSCLC) patients. [10–12]. The Mst/Yap pathway regulates organism growth and is very conservative in both human and *Drosophila* [13–16]. The oncoprotein Yap negatively regulated by the tumor suppressor Mst in this kinase cascade transcriptionally activates its downstream target genes involved in cell growth and survival [17–21]. The upstream signal molecules phosphorylate and activate Mst, one of the core suppressor components inside the hippo pathway. Sav (WW-45) is another core component of the Mst tumor suppressor pathway, offering assistance of the activated Mst kinase phosphorylates and activates Lats, which itself phosphorylates Yap with the involvement of the Mob. Phosphorylated Yap is retained in the cytoplasm by interacting with 14-3-3 protein, so cannot combine with its target transcription factors to interact with promoters, and is thereby inactivated [19–25]. However, in the case of the loss of function of the Mst cell signals, the unphosphorylated Yap gets into the nucleus and physically binds to the transcription factors TEAD family proteins, which itself regulates the target genes of the Mst/Yap pathway such as connective tissue growth factor (CTGF), Cyr61, Cyclin D1, Survivin, and so on [25–31].

Norcantharidin (NCTD) has been applied in cancer treatment in China for years. NCTD is a demethylated form of cantharidin that has an important anticancer role in breast cancer, liver cancer, gallbladder carcinoma, prostate cancer, mantle cell lymphoma, hepatocellular carcinoma, leukemia, and colon cancer with fewer side effects [32–37]. NCTD has been found to induce both *in vitro* and *in vivo* inhibitions in growth of a variety of human tumor cells via modulating functions of cell cycle kinases leading to tumor cell cycle arrests. NCTD also can activate the mitochondrial pathway and ROS accumulation to induce tumor cell apoptosis [38–39] and significantly reduce the tumor angiogenesis [40–42] However, the underlying mechanisms by which NCTD inhibits lung cancer progression and metastasis are still largely undefined.

This study examined the specificity of NCTD in repressing YAP to inhibit non-small cell lung carcinoma progression and revealed that NCTD inhibits cell growth and metastasis, enhances s cell apoptosis and senescence, and arrest cell cycle in lung cancer cells via specifically downregulating aberrantly activated YAP signaling. Our results suggested that NCTD is a novel therapeutic drug candidate for NSCLC which specifically and sensitively target YAP signal pathway.

## RESULTS

### Aberrant activation of YAP in lung cancer specimens

**Figure 1:**
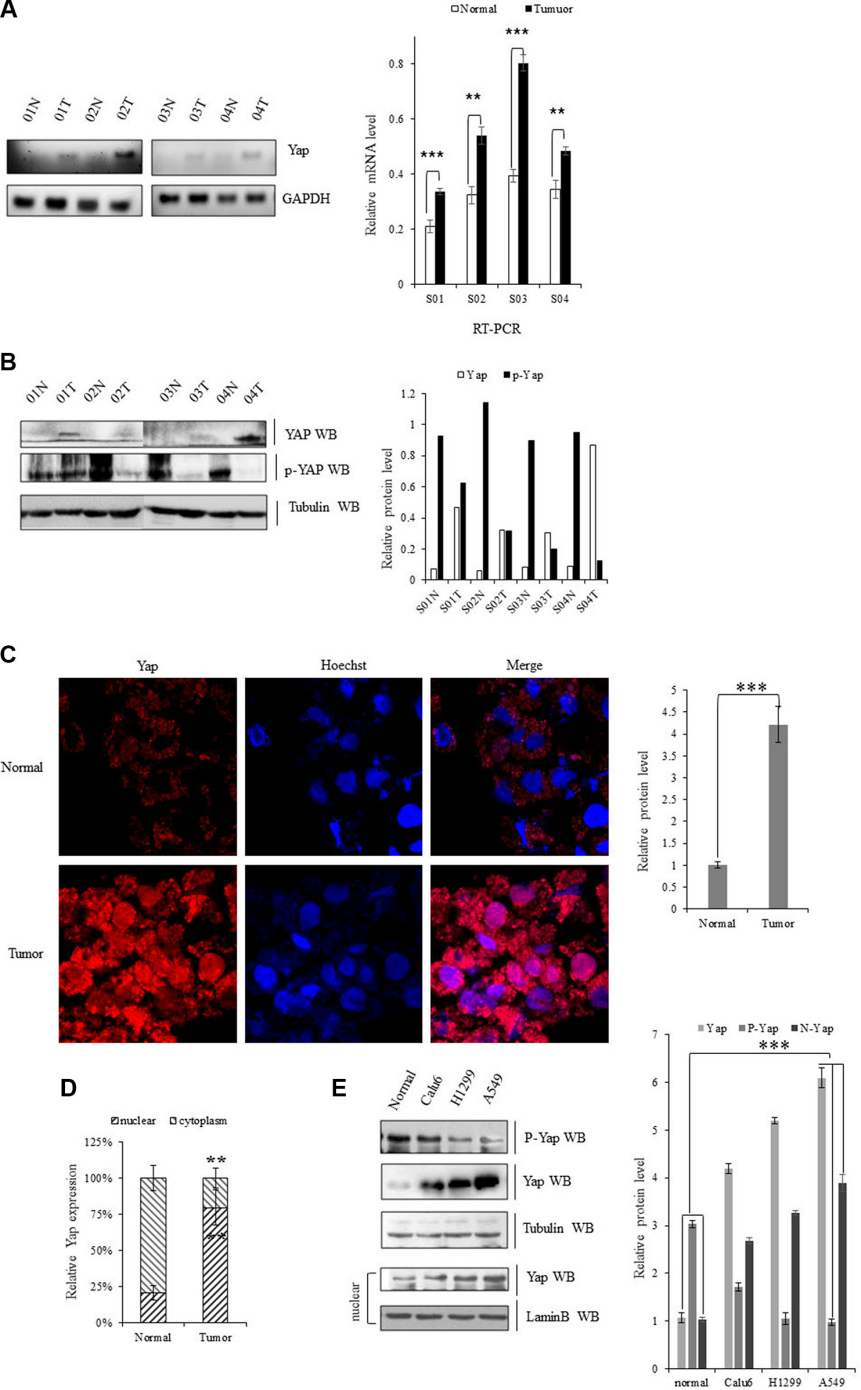
Upregulation of YAP in lung cancer cells (**A**) Gel-based RT-PCR with densitometric quatitation demonstrating elevated expression of YAP in human NSCLC tissues compared with their normal adjacent lung tissues. (**B**) Immunoblotting with densitometric quantitation demonstrating increased protein level of Yap and decreased p-YAP in human NSCLC tissues compared with their normal adjacent lung tissues. (**C**, **D**) Immunofluorescent staining of Yap proteins showing increased YAP accumulated in nuclear in NSCLC samples compared with their normal adjacent lung tissues while more YAP was localized in cytoplasm of those normal adjacent lung tissues. (**E**) Immunoblotting with densitometric quantitation demonstrating increased protein level of total Yap as well as nuclear Yap and decreased p-YAP in Calu-6, H1299, and A549 cells than normal cell line. **P < 0.001 ***P < 0.0001 by Student's *t*-test

For examination of the expression of YAP in human lung tumor tissues, we performed both RT-PCR and immunoblotting and found that mRNA levels of Yap were higher in lung tumor tissues compared with their adjacent normal lung tissues in four pairs of specimens (Figure 1A). As indicated in Figure 1B, the total YAP protein levels were also significantly elevated in all four lung tumor tissue compared to their adjacent normal lung tissues. Meanwhile, the phosphorylated Yap (cytoplasm part) was lower in these lung tumor samples than these adjacent normal lung tissues. Immunofluorescent staining of Yap proteins further showed more accumulated YAP in nuclear of NSCLC samples relative to their normal adjacent lung tissues while more p-YAP was localized in cytoplasm of those normal adjacent lung tissues (Figure 1C, 1D). Endogenous Yap protein level was also significantly higher in Calu-6, H1299, and A549 cells than normal cell line (Figure 1E).

### Yap knockdown inhibits cell growth and invasion

**Figure 2:**
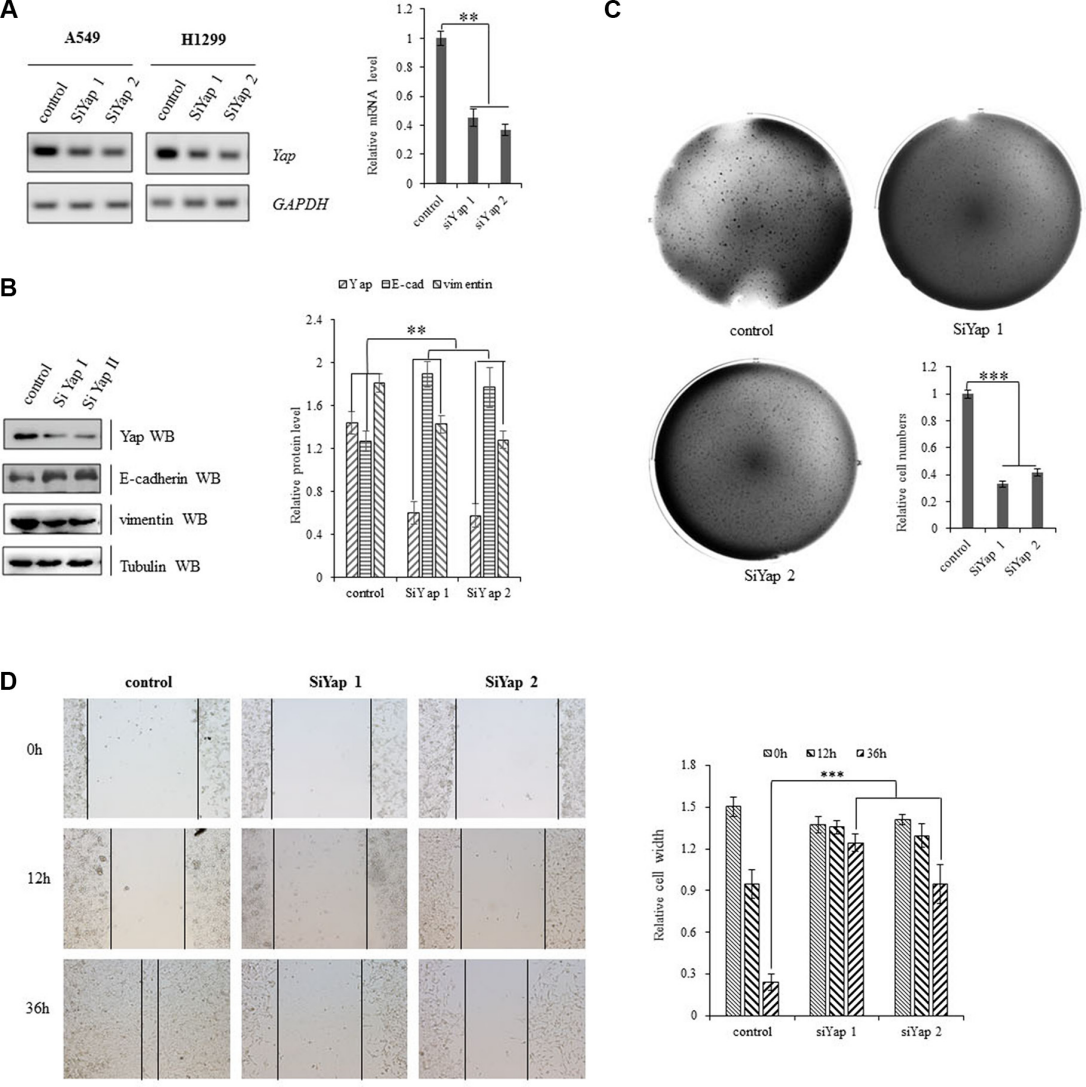
Activation of YAP drives NSCLC progression (**A**) Knockdown of YAP decreased transcription activity of YAP in A549 and H1299 cells as shown in Gel-based RT-PCR with densitometric quantitation. (**B**) Immunoblotting with densitometric quantitation demonstrating decreased YAP and Vimentin protein expressions and increased E-cadherin protein level in A549 cells with siRNA-YAP treatment. (**C**) Knockdown of Yap arrested clonal formation to delay the clonogenecity of A549 cells. (**D**) Scratch test showing that knockdown of Yap significantly decreased scar healing rates at 36 hours after siRNA-YAP treatment in A549 cells. **P < 0.001 ***P < 0.0001 by Student's *t*-test.

Specific silencing YAP by siRNA was applied to explore whether activation of the oncogene YAP underlies the initiation, progression, and metastasis of lung cancer cells. Knockdown of YAP increased E-cadherin protein and mRNA levels and decreased the protein level of vimentin in A549 cells (Figure 2A, 2B). Depletion of YAP also significantly decreased clonal formation (Figure 2C) and cell migration (Figure 2D) of A549 cells, which indicates the critical role of YAP in invasive growth of NSCLC cells.

### NCTD represses the activity of YAP in the lung cancer cells

**Figure 3:**
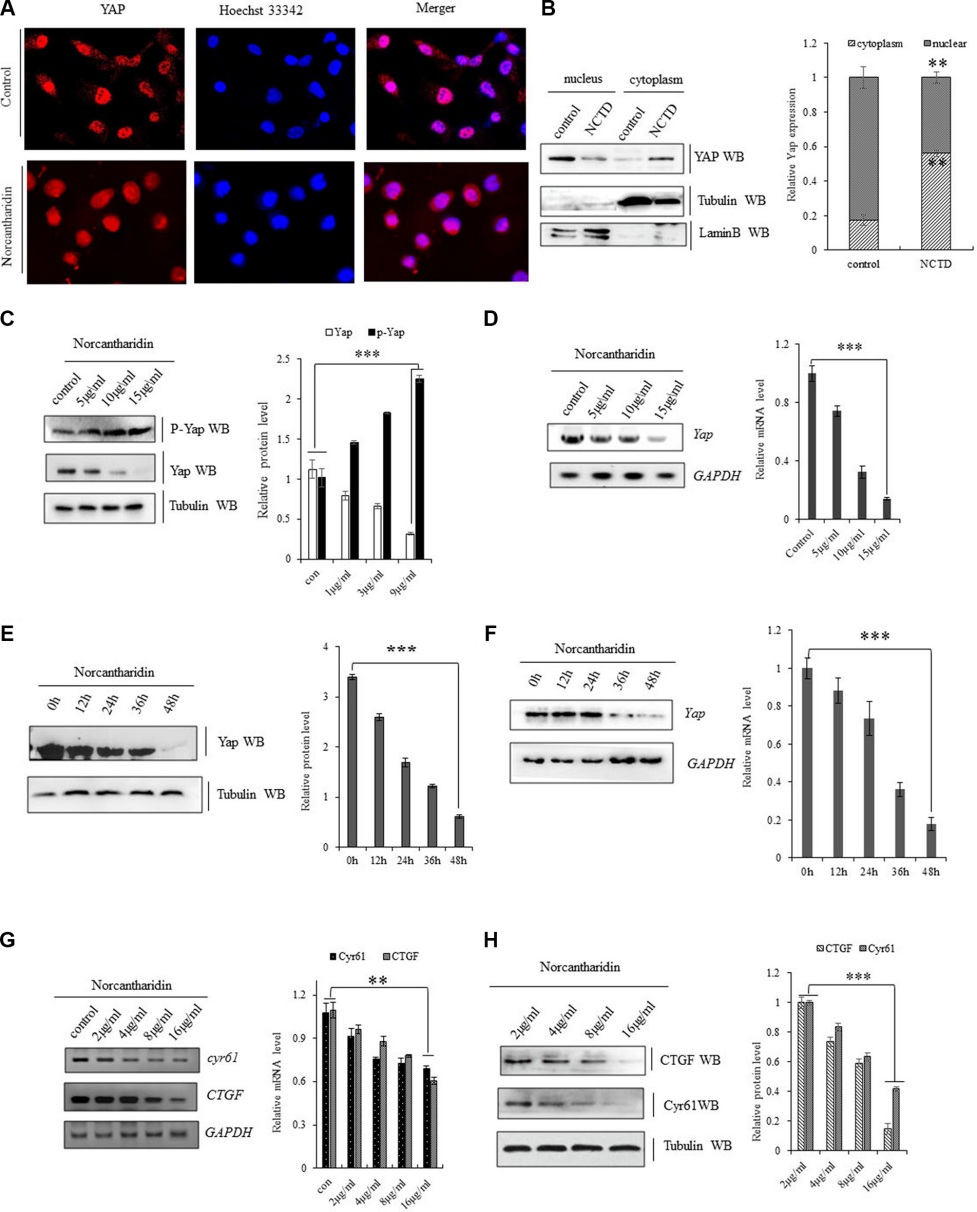
NCTD represses YAP signaling pathway (**A**) Immunofluorescent staining of Yap proteins demonstrating that NCYD treatment at 8 μM for 36 hours enhanced the translocation of Yap from nuclear to cytoplasm in A549 cells. (**B**) Immunoblotting showing decreased YAP in nuclear and relatively enriched inside cytoplasm in A549 cells treated with NCTD at 8 μM for 36 h. (**C**, **D**) NCTD dose-dependently reduced Yap mRNA(C) and protein level (D, G), but increased phosphorylated YAP (D) in A549 cells for 72 hours. (**E**, **F**) NCTD time-dependently reduced Yap mRNA(E) and protein level (F) in A549 cells at 15 μΜ. (**G**, **H**) NCTD dose-dependently decreased CYR61 and CTGF at both mRNA(G) and protein (H) level from 2 μM to 16 μM in A549 cells for 72 hours. **P < 0.001 ***P < 0.0001 by Student's *t*-test.

Norcantharidin (NCTD) was further mechanistically explored in its interfering YAP signal pathways in lung cancer cells. NCTD at 8 μM for 36 h decreased YAP in nuclear and enriched phoshorylated YAP inside cytoplasm leading to the translocation of Yap from nuclear to cytoplasm in A549 cells (Figure 3A, 3B). We also found that NCTD dose-dependently (Figure 3C, 3D, 3G) and time-dependently (Figure 3E, 3F) reduced Yap mRNA and protein level, but increased phosphorylated YAP in A549 cells and H1299 cells (Supplementary Figure S1). Moreover, immunofluorescence analysis indicates that the protein level of Yap was obvious reduced in A549 cells treated with 15 μM NCTD for 72 h (Supplementary Figure S2A). Since NCTD induced repression of YAP in NSCLC cells, we further examined the effect of NCTD on YAP downstream signaling pathway. Both RT-PCR and immunoblotting showed NCTD inhibited YAP and then consequently decreased YAP downstream targets CYR61 and CTGF at both mRNA (Figure 3G) and protein (Figure 3H) levels from 4 μM to 16 μM in A549 cells for 72 hours in dose-dependent manner. Additive immunofluorescent staining of those YAP downstream targets semi-quantitatively demonstrated that NCTD at 4 μM and 16 μM for 72 hours significantly decreased both CTGF (Supplementary Figure S2B) and CYR61(Supplementary Figure S2C) protein level in A549 cells.

### NCTD interferes the YAP-mediated NSCLC cell proliferation

**Figure 4:**
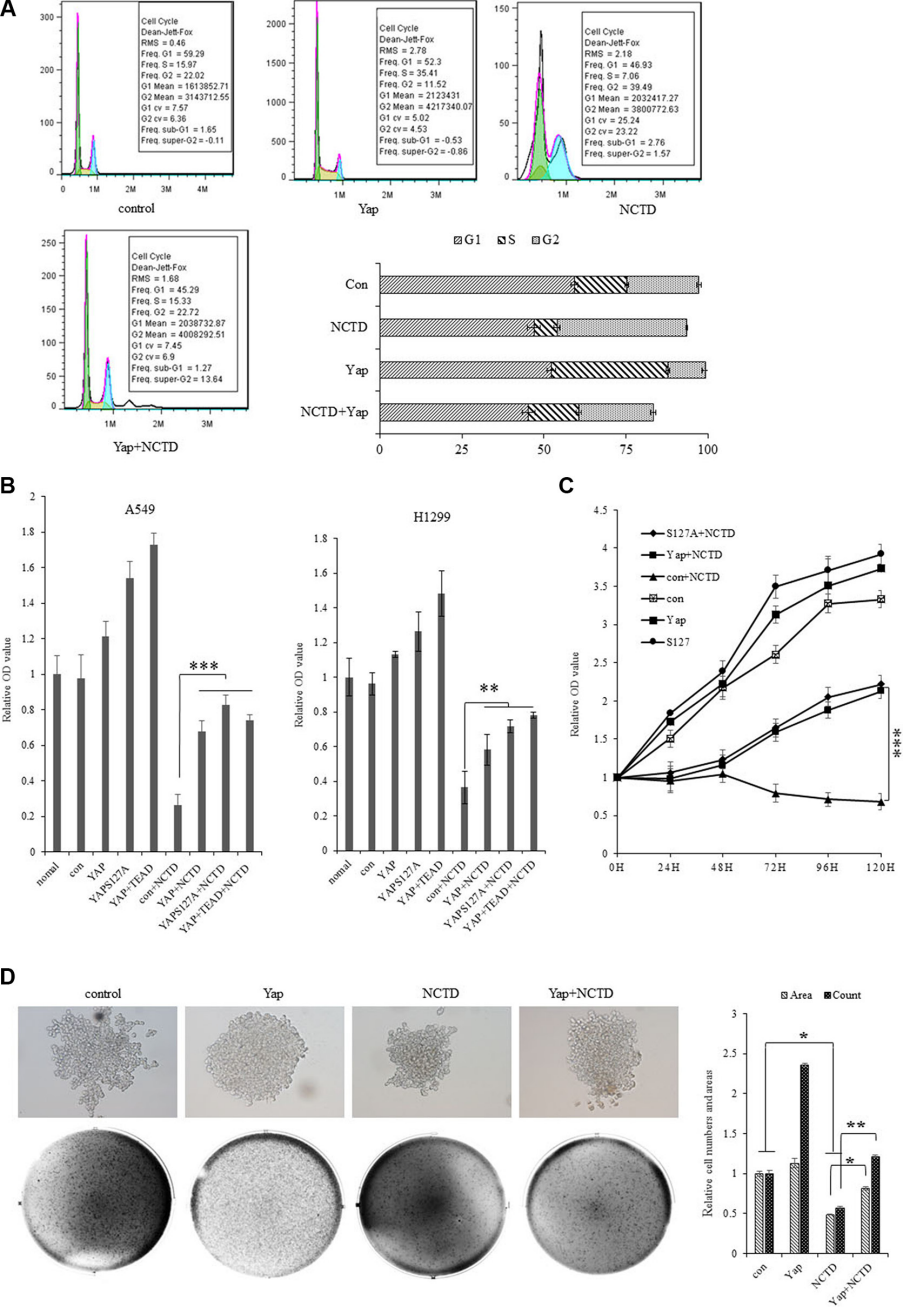
NCTD interferes the YAP-mediated NSCLC cell proliferation (**A**) Treatment of NCTD at15 μM for 72 h induced significant G2 arrests and Yap blocks the effect of NCTD in A549 cells as showing in both representative histograms of cell cycle distribution and their quantitation analysis. (**B**) *In vitro* proliferation assay demonstrating that NCTD at 15 μM significantly arrested cellular proliferation of A549 and H1299 cells with or without stably expressing YAP, YAPS127A, YAP and TEAD for 72 hours. (**C**) Cell growth assay showing that NCTD at 15 μM time-dependently and significantly arrested cellular proliferation of A549 cells with or without stably expressing YAP, YAPS127A, YAP and TEAD. (**D**) Soft agar colony formation assay demonstrating that ectopic expression of Yap significantly enhanced colony formation density which was blocked by NCTD at 15 μM for 72 hours in H1299 cells. ***P < 0.0001 by Student's *t*-test.

To investigate the potential biological function of NCTD in NSCLC cells, our examination of cell cycle profile in lung cancer cells treated with NCTD revealed that treatment of NCTD at15 μM for 72 h induced significant G2 arrests in A549 cells and dramatically blocked the YAP-induced S introduction as showed in both representative histograms of cell cycle distribution and their quantitation analysis (Figure 4A). As in Figure 4B, 4C, *in vitro* proliferation assay demonstrated that ectopic expression of YAP, or its variant YAPS127A, or YAP plus its downstream component TEAD significantly stimulated A549 and H1299 cells in growth and NCTD at 15 μM time-dependently and significantly arrested the proliferation rates of A549 and H1299 cells with or without stably expressing YAP, or YAPS127A, or YAP plus TEAD. Furthermore, soft agar colony formation assay was performed and demonstrated that ectopic expression of Yap significantly enhanced colony formation density which was blocked by NCTD at 15 μM for 72 hours in H1299 cells (Figure 4D).

### YAP rescues the NCTD -mediated lung cancer cell senescence and apoptosis

**Figure 5:**
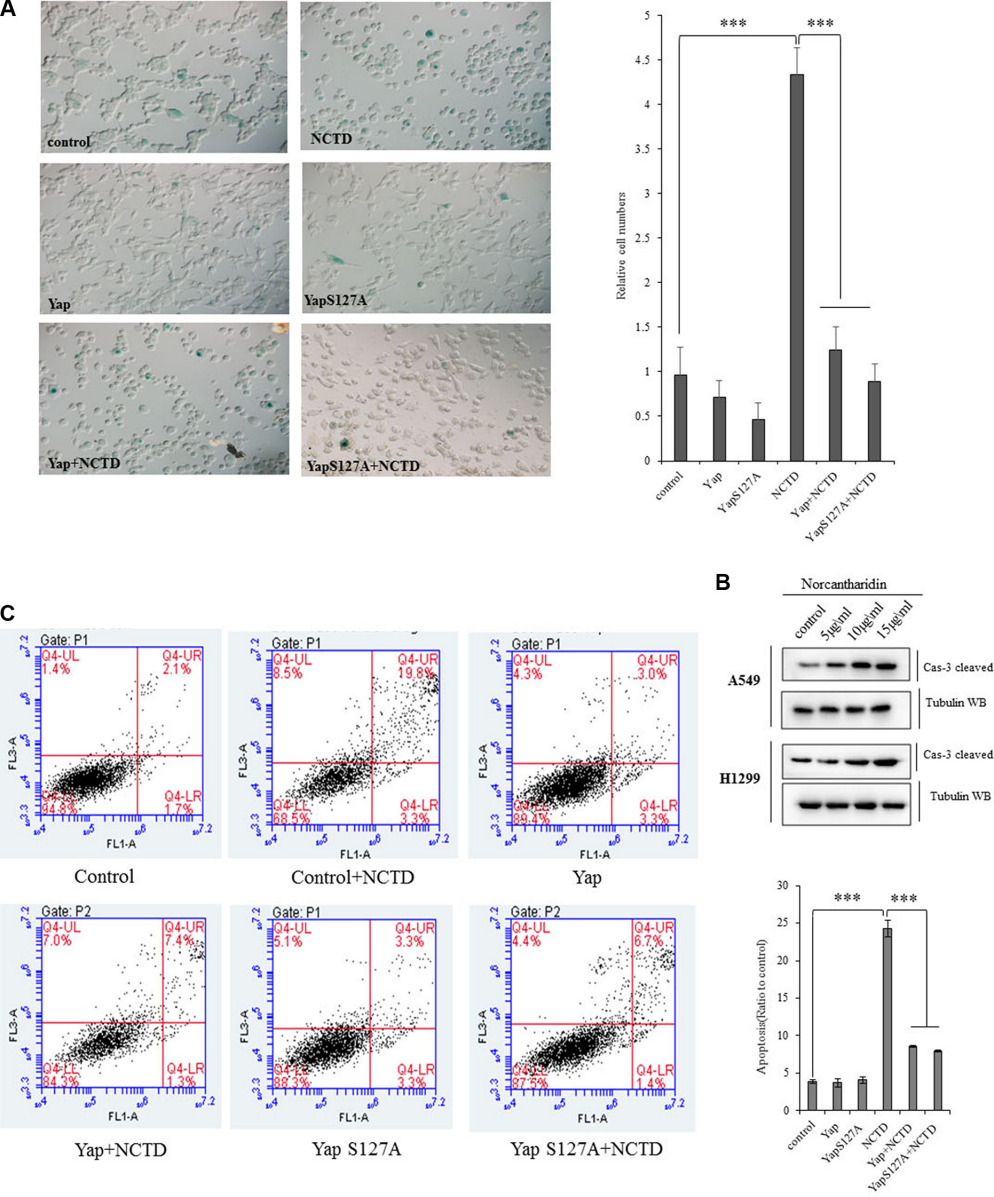
Yap blocks the NCTD-mediated NSCLC cell senescence and apoptosis (**A**) SA-β-Gal assay showing that NCTD at 15 μM significantly increased cell senescence phenotype in A549 cells for 72 h, in which ectopic expression of YAP, YAPS127A, YAP and TEAD completely blocked NCTD-induced senescence enhancement. (**B**) Flow cytometric assay demonstrating that treatment of NCTD at 15 μM significantly induced apoptosis in A549 cells for 72 h, in which ectopic expression of YAP, YAPS127A, YAP and TEAD partially blocked NCTD-induced apoptosis. (**C**) Immunoblotting showing that NCTD dose-dependently increased pro-apoptosis protein caspase-3 in A549 and H1299 cells for 72 hours. ***P < 0.0001 by Student's *t*-test.

Cellular senescence and apoptosis resistance are the death-avoiding mechanism of tumor cells. It has been reported that Yap represses senescence in human tumor cells [43, 44]. To examine whether NCTD interferes YAP-induced cellular senescence arrests in lung cancer cells, the senescence assay was performed and demonstrated that NCTD at 15 μM significantly increased cell senescence phenotype in A549 cells for 72 h, in which ectopic expression of YAP, or YAPS127A, or YAP plus TEAD completely blocked NCTD-induced senescence enhancement (Figure 5A), and our flow cytometric assay also showed that treatment of NCTD at 15 μM significantly induced apoptosis in A549 cells for 72 h, in which ectopic expression of YAP,orYAPS127A, or YAP plus TEAD at least partially blocked NCTD-induced apoptosis (Figure 5B). Additional immunoblotting confirmed that NCTD dose-dependently increased pro-apoptosis protein caspase-3 in A549 and H1299 cells for 72 hours (Figure 5C).

### NCTD interferes the Yap-mediated NSCLC cell invasiveness and EMT

**Figure 6:**
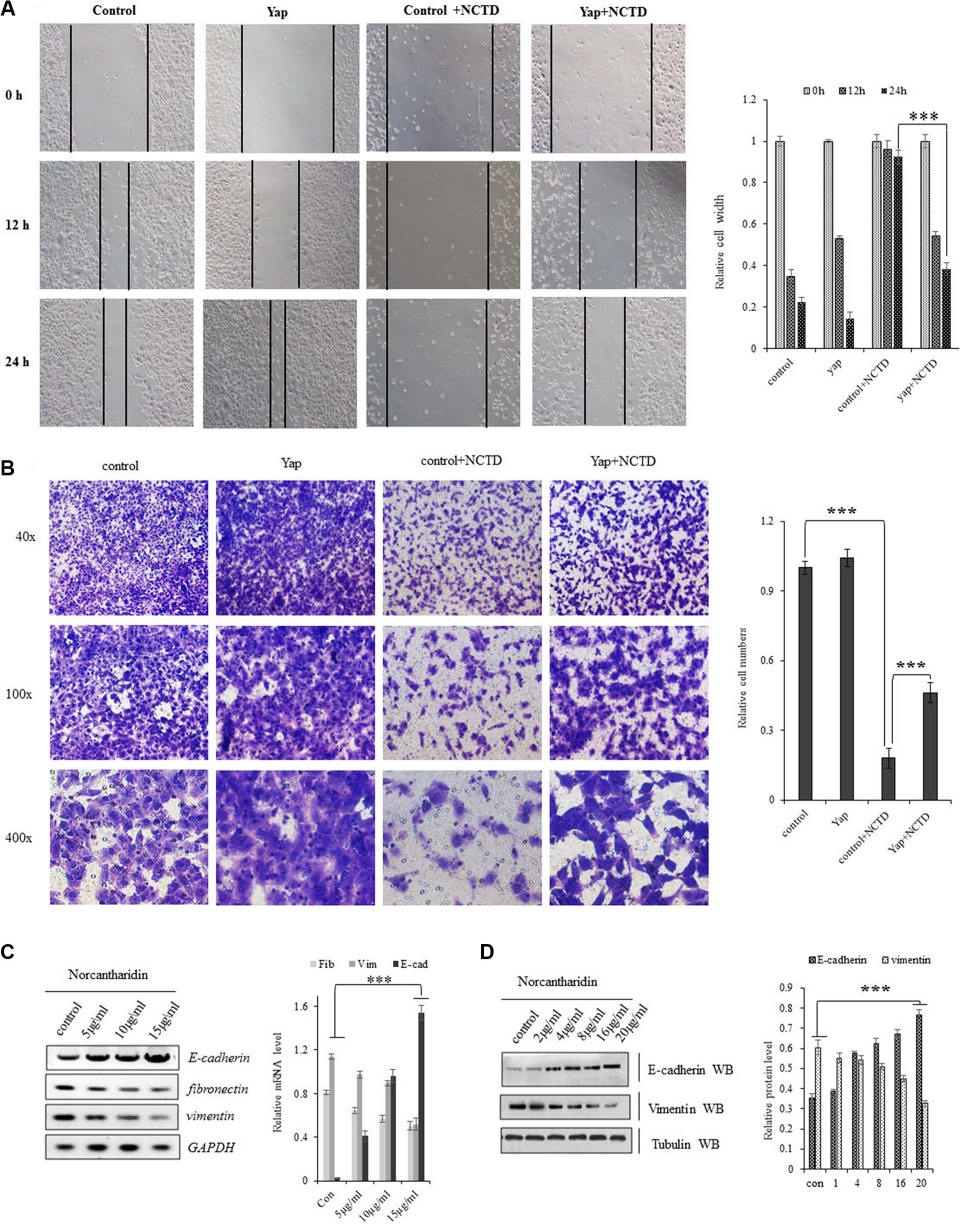
NCTD interferes the Yap-mediated NSCLC cell invasiveness and EMT (**A**) Scratch assay showing treatment of NCTD at 15 μM for 12–24 hours dramatically decreased Yap-induced enhancement in A549 cell migration. (**B**) Matrigel invasion assay identified that treatment of NCTD at 15 μM for 72 hours dramatically decreased cell invasive growth and overexpressing YAP partially blocked NCTD-induced inhibition in cell invasive growth. (**C**, **D**) NCTD dose-dependently increased E-cadherin and increased fibronectin and vimentin in mRNA(C) and protein (D) level in A549 cells for 72 hours. ***P < 0.0001 by Student's *t*-test. **P < 0.001 ***P < 0.0001 by Student's *t*-test.

Cellular invasiveness are the most distinguished features of tumor cells. In scratch assay (Figure 6A), we found that treatment of NCTD at 15 μM for 12–24 hours dramatically decreased Yap-induced enhancement in A549 cell migration. For examining invasive growth of those lung tumor cells, the matrigel invasion assay identified that treatment of NCTD at 15 μM for 72 hours dramatically decreased cell invasive growth and overexpressing YAP partially blocked NCTD-induced inhibition in cell invasive growth (Figure 6B).

Since NCTD can inhibit the Yap-mediated invasive growth of lung cancer cells, we further checked whether NCTD interferes the YAP-mediated cellular phenotype switching in lung cancer cells by examining the EMT hall marks such as E-cadherin, Vimentin and Fibronectin. As in Figure (6C, 6D), NCTD dose-dependently enhanced E-cadherin and represses fibronectin as well as vimentin in mRNA(Figure 6C) and protein (Figure 6D) level in A549 cells for 72 hours, which suggested that NCTD interferes the YAP-mediated EMT in NSCLC cells.

## DISCUSSION

Our study explored the specificity and efficacy of norcantharidin (NCTD) in inhibiting NSCLC progression and defined it as a novel therapeutic drug candidate for NSCLC which specifically and sensitively target YAP signal pathway.

Although substantial improvements in therapeutic interference and early diagnosis of lung cancer have increased survival ability and life qualities of lung cancer patients over recent decades, it still remains the most aggressive malignant tumor with one of the lowest survival rates. To date, almost all NSCLC patients with advanced and unresectable disease have few choices in treatments except chemotherapy and radiotherapy, which bring uncontrollable side effects. Therefore, it is in great demands now to identify and characterize novel selective drug candidates with none or low toxicities in lung cancer therapies.

The potential of NCTD in cancer treatment was explored as early in 1980s in China [5]. NCTD not only can inhibit the proliferation of varieties of cancer cell lines and those *in vivo* xenografts but also present no side effects both *in vitro* and *in vivo* [24, 25]. Applying NCTD as a monotherapeutic drug in clinical trials substantially benefited patients with different human malignancies [6, 7]. Our results confirmed that NCTD treatment significantly arrested the proliferation of A549 and H1299 cells in dose- and time-dependent manners via dose-dependent repression of activities of YAP and its downstream target genes, which eventually leads to the cell cycle arrests in lung cancer cells. In addition, NCTD induced dramatic reduction in invasive growth of A549 cells implying its potentials in targeting lung cancer metastasis. Our findings strongly support the development and application of NCTD as a novel therapeutic option in the treatment of human lung cancer.

The efficacy of available lung cancer therapeutic interference is significantly limited by various resistance mechanisms to those drugs. YAP is one of the novel anti-cancer drug targets involved in chemo-resistance in lung cancer. Activation of the oncogene YAP underlying the initiation, progression, and metastasis of lung cancer associates with poor prognosis and may promote metastatic spread and confer drug resistance against targeted therapy. Given the selective induction of tumor cell apoptosis by NCTD without affecting normal cells [24–26] and highly activation of YAP in lung cancer, it is reasonable to test the therapeutic efficacy of NCTD in human lung cancer treatment and its specificity derived from the selective YAP targeting. Our study revealed that YAP signal pathways were aberrantly activated in lung cancer tissues and cells which conferred more proliferative and invasive phenotypes to human lung cancer cells. We confirmed that NCTD specifically repressed YAP signaling pathway to interfere the YAP-mediated NSCLC progression and metastasis via arresting cell cycle, apoptosis and senescence. We also found NCTD-mediated repression of YAP decreased epithelial-to-mesenchymal transition (EMT) and reduced the motile and invasive cellular phenotype and the metastatic prowess *in vitro* via enhancing E-Cadherin and decreasing fibronectin/vimentin.

Given lacking extensive molecular dissection of mechanisms governing downregulation of YAP by NCTD, we further examined the NCTD-induced alterations in YAP expression (mRNA and protein) and post-translational modification (phosphorylation) in lung cancer cells. Our results indicated that NCTD not only significantly and selectively represses transcriptional activity of YAP, it also enhances the phosphorylation of YAP to facilitate the cytosol translocation of YAP from nuclear in lung cancer cells (Figure 3A, 3B). We first found that NCTD dose-dependently (Figure 3C, 3D) and time-dependently (Figure 3E, 3F) reduced Yap mRNA and protein level, but increased phosphorylated YAP in A549 cells, which may suggest the rationale for the molecular mechanism in NCTD's non-resistance therapeutic function for lung cancer.

While our results characterized the anti-cancer functions of NCTD in preventing NSCLC progression and metastasis, we have not defined the role of NCTD in the unavoidable chemoresistance development in lung cancer treatment. Aberrantly activated YAP not only renders the priority of lung cancer cells in their proliferation and metastatic colonization, but also confers the drug resistance in NSCLC cells. Although we did not address this issue in this report, the investigation of overcoming the YAP activation-induce drug resistance by NCTD and other analogs is undergoing.

Collectively, NCTD can function as an efficient non-resistance therapeutic drug for NSCLC patients via inhibiting the activation of YAP to increase the apoptosis and senescence, and arresting tumor cell proliferation. Notable, we demonstrated that these effects of NCTD were repressed by transient transfection of Yap. This study verified and proposed that NCTD has all the potentials to be selected as a chemotherapeutic candidate for repressing tumorigenesis and the development of NSCLC via selectively targeting YAP. NCTD interrupted the cell cycle progression and rewired cell proportion distribution among all the cell cycle phases leading to the cell growth arrest in NSCLC cells. Finally, exceptional growth and metastasis inhibition by NCTD in our study grants any further clinical investigation regarding its anti-tumor effects in NSCLC patients.

## MATERIALS AND METHODS

### Cell lines and treatment conditions

All lung cancer lines (A549, H1299 and Calu6) were obtained from American Type Culture Collection (ATCC; Manassas, VA), and maintained in RPMI media supplemented with 10% FBS (FBS; Hyclone, USA), 10 mM of glutamic acid, and 1% penicillin/streptomycin (normal media). Cells were subcultured as necessary, and harvested at various time-points for analysis.

### Human tissues

All the human lung cancer and normal lung specimens were collected in Affiliated Hospital of Binzhou Medical College with written consents of patients and the approval from the Institute Research Ethics Committee. All tissues were immediately snap-frozen with a portion of harvested tissue sent for immediate histologic confirmation by an independent, anatomic pathologist in a blinded manner. A total of 10 human lung cancer samples with paired pathologically normal lungs were used for real-time PCR analysis and another 10 lung cancer were used for WB analysis.

### Plasmid constructs for over-expression

cDNA overexpressing constructs for Flag-tagged Yap, Yap S127A and Myc-tagged TEAD2 were made from the pcDNA3.1 vector (Invitrogen). Plasmid constructs (2 μg) were transfected into cells using Lipofectamine 2000 (Invitrogen, Carlsbad, CA), followed by analysis 48–72 h later.

### Immunofluorescent staining

For analysis of Yap subcellular localization and the protein levels of Yap, Cyr61 and CTGF, A549 and H1299 cells were grown on coverslips in a 24-well plate overnight and after 24h, treated with NCTD. After 36 h, cells were fixed in 4% formaldehyde for 30 min and permeabilized by incubation in 3% BSA in PBS for 30 min. The coverslips were subsequently incubated with rabbit anti-Yap (CST, #8418), Cyr61(Abcam, 24448) and CTGF (Abcam, 6992) monoclonal at 1:2000 dilution in PBS containing 3% BSA. Alex Fluor AF 594 (red) anti-rabbit monoclonal secondary fluorescence antibodies at 1:1000 dilution in PBS containing 3% BSA. DAPI (3 μg/mL) was used for nuclear staining. Images were obtained with Zeiss Axio Imager Z1 Fluorescent Microscope.

### Cell cycle and Annexin V staining and flow cytometry

For cell cycle analysis, drug-treated cells with 80% confluence were harvested and fixed with 70% ethanol. Then cells were taken for PI staining and cell cycle was analyzed using flow cytometry. For apoptosis analysis, cells were cultured in attachment then trypsinized and stained with PI/Annexin V (Vazyme, Apoptosis Detection Kit). Data were collected and analyzed on a BD FACSC and using FACSD via software.

### RNA isolation and RT-PCR assay

Total RNA was isolated using Trizol reagent (TransGen Biotech) and retro-transcribed into first-strand cDNA using TransScript All-in-One First-Strand cDNA Synthesis (TransGen Biotech). cDNAs were subjected to reverse PCR assay corresponding primer. GAPDH (human) served as internal control. The reverse PCR primers as follows:

Yap forward primer: GGACCCCAGACGACT TCCTCAACAG

Yap reverse primer: CCTTCCAGTGTGCCAAGG TCCACAT

E-cadherin forward primer: ACCATTAACAGGAA CACAGG

E-cadherin reverse primer: CAGTCACTTTCAGTG TGGTG

Vimentin forward primer: CGCCAACTACATCG ACAAGGTGC

Vimentin reverse primer: CTGGTCCACCTGCC GGCGCAG

Fibronectin forward primer: CCTGAGGATGGAAT CCATGAGC

Fibronectin reverse primer: GGCTCTCCATATCGT GCAAG

### Western blot analysis

Protein extracts from tissues and cells were lysed in NP-40 buffer (150 mM NaCl, 1% Triton X-100, 10 mM Tris pH 7.4, 1 mM EDTA pH 8.0, 1 mM EGTA pH 8.0, 0.5% NP-40, and 1 mM PMSF) at 25°C for 30 min. The supernatants of the samples were diluted by 5× loading, followed by Western blotting as previous reports. Samples were separated on NuPAGE 4–12% Bis-Tris gels (Invitrogen) and blotted onto Immobilon P membrane (Millipore), and proteins detected using enhanced chemiluminescence detection reagents (Amersham). Antibodies used for western analysis were anti-Yap, anti-pYap and tubulin antibody (Cell Signaling Technology 1:1000), anti-E-cadherin, anti-vimentin, capase-3 cleave (Abcam 1:1000), and laminB (proteintech, 1:2000)

### SA-β-gal staining

Senescence-associated β-galactosidase (SA-β-gal) was detected using Senescence β-Galactosidase Staining Kit (Beyotime, C0602) following the manufacturer's protocol.

### CCK-8 analysis

Dispense 100 μl of cell suspension (5000 cells/ well) in a 96-well plate. Pre-incubate the plate for 24 hours in a humidified incubator at 37°C, 5% CO_2_. Then add 15 μM NCTD to the tested plate. Incubate the plate for 72 hours in the incubator. Add 10 μl of CCK-8 (C0037, beyotime) solution to each well of the plate and then incubate the plate for 4 hours in the incubator. Measure the absorbance at 450 nm using a microplate reader.

### Wound-healing assay

Cells were plated in 6-well culture plate and incubated in 5% CO_2_ at 37°C for 24 h. Single layer confluent cells were wounded by scraping using 200P micropipette tip, then washed by PBS and incubated in RPMI containing 2% FBS with various 15 μg/ml of NCTD and relevant plasmid for different times. The result of this experiment was analyzed by the Olympus light microscope.

### Transwell migration assay

Transwell migration assay was carried out with a 24-well chamber (Costar 3422, Corning Inc., Corning, NY). The lower and upper chambers were separated by a polycarbonate membrane (8 μm pore size). Cells (1 × 10^5^) were resuspended in RPMI medium containing free FBS in the upper chamber. The RPMI medium containing 10% FBS was added to the lower chamber. A549 cells were allowed to migrate for 36 h at 37°C in a humidified atmosphere containing 5% CO_2_. The membrane was fixed in methanol for 20 min at 4°C, and then stained with crystal violet. Cells on the upper side of the membrane were removed by PBS-rinsed cotton swabs. Cells on the lower side of the membrane were counted under an Olympus light microscope.

### Colonogenecity functional assay

For soft agar colony formation assay, virus-infected cells were added to growth medium with 0.2% agar and layered onto 1% agar beds in six-well plates. Cells were fed with 1 ml of medium every three days. The colonies were stained with 0.005% crystal violet and counted in 2–3 weeks.

### Proliferation/viability assay by MTT

Cell viability was determined by 3-(4,5-dimethylthiazol-2yl)-2,5-diphenyltetrazolium bromide (MTT) assay in 96-well plates, as previously described. Cells were incubated with 15 μM NCTD and indicated plasmids for 72 h followed by MTT for 4 h, and then 100 μL DMSO (Dimethyl Sulphoxide) was added to dissolve the formazan crystals. The absorbance was read at 570 nm using a spectrophotometer (Synergy H1, BioTek). Cell viability was calculated as the relative absorbance compared to DMSO vehicle control absorbance.

### Novelty and impact

This studies are the first to examine specific targeting YAP signaling by NCTD in human lung cancer treatment, and clearly suggest that activation of the oncogene YAP is involved in the initiation, progression, metastasis, and drug resistance against targeted therapy of lung cancer and can be significantly repressed by NCTD as monotherapeutic agent. Our studies provide a direct mechanistic link between NCTD and its molecular targets in YAP signal pathways in lung cancer therapy.

## SUPPLEMENTARY MATERIALS FIGURES AND TABLES



## Abbreviations

NCTD: Norcantharidin
NSCLC: Non-small cell lung carcinoma
EMT: Epithelial-to-mesenchymal transition
CTGF: Connective tissue growth factor
