# Supplementary material for: Repression of YAP by NCTD disrupts NSCLC progression
Source: Oncotarget. 2016 Nov 26;8(2):2307–19. doi: 10.18632/oncotarget.13668 (PMC5356801; doi:10.18632/oncotarget.13668)
Supplement: Supplementary file 1 [file oncotarget-08-2307-s001.pdf]

Repression of YAP by NCTD disrupts NSCLC progression

Supplementary Materials

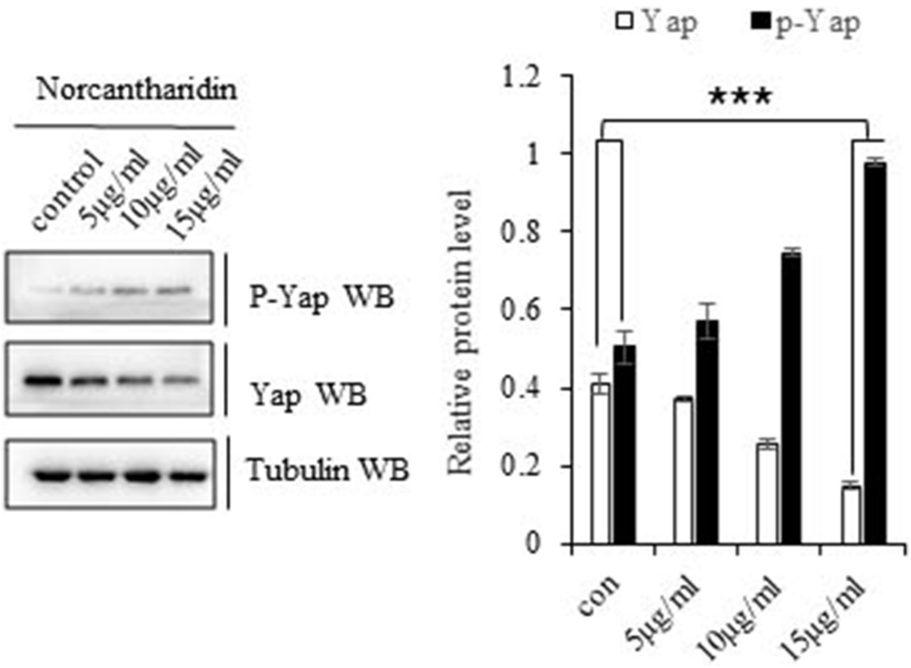

Supplementary Figure S1: NCTD dose-dependently reduced Yap protein level, but increased phosphorylated YAP in the H1299 cells.

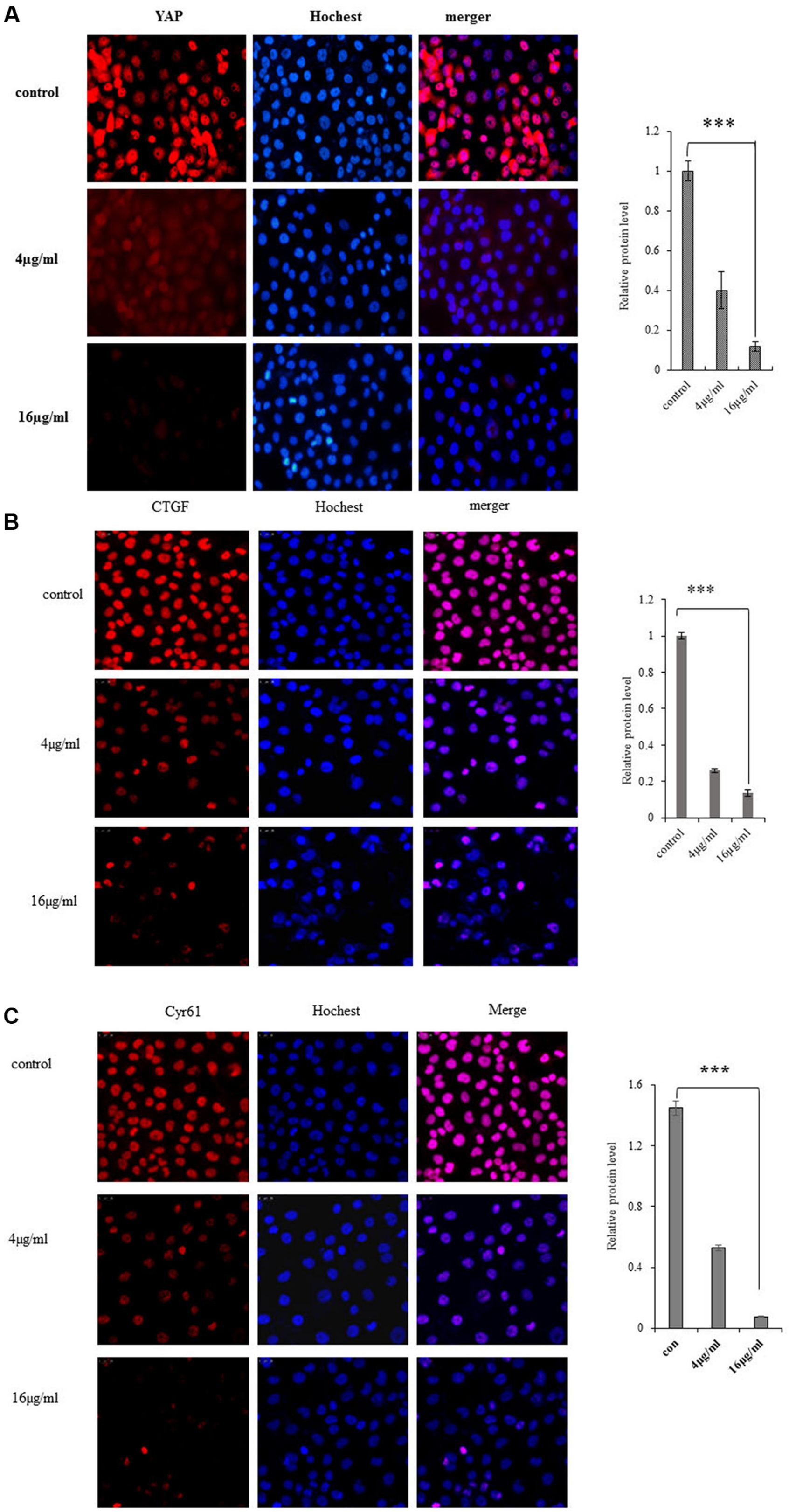

Supplementary Figure S2: NCTD represses YAP and its downstream targets. (**A**–**C**) Representative immunofluorescent staining of Yap (A), CTGF (B) and CYR61 (C) proteins with semi-quantitation showing that NCTD at 4 μM and 16 μM for 72 hours significantly decreased both CTGF (B) and CYR61 (C) protein level in A549 cells. ***$P < 0.0001$ by Student's *t*-test.
